# METFORMIN TO TARGET FRAILTY IN OLDER ADULTS

**DOI:** 10.1093/geroni/igaf122.1104

**Published:** 2025-12-31

**Authors:** Sara Espinoza, Nicolas Musi

**Affiliations:** Cedars-Sinai Medical Center, Los Angeles, California, United States; Cedars-Sinai Medical Center, Los Angeles, California, United States

## Abstract

It has been postulated that metformin may extend healthspan, but no long-term trials have been done in humans. We conducted a randomized, double-blinded, placebo-controlled clinical trial of metformin to reduce frailty in older adults (≥65 years) with glucose intolerance. Participants who were frail at baseline (Fried criteria) were excluded. Participants were randomized to 24 months of metformin (initiated at 500 mg/day and titrated to maximum tolerated dose up to 2,000 mg/day) or matching placebo. All participants received one session of diet and exercise counseling prior to initiation of study drug. The primary outcome was frailty as measured by the frailty index based on deficit accumulation model and frailty phenotype based on Fried criteria, which was assessed every 6 months. We applied generalized estimating equations (GEE) models to examine the change in frailty per month by frailty index and Fried criteria. 145 participants were randomized; intention to treat analysis included 141 participants who took at least one dose of study drug. At baseline, participants were 48% female, 35% Hispanic/Latino, and mean age was 71.8 ±5.3 years. Metformin did not cause serious adverse events. Metformin led to a significant reduction in frailty progression rate per month assessed by frailty index compared to placebo (-0.0494 ±0.0216, 95%CI: -0.0918, -0.0071, p=0.0222). No difference was observed by Fried criteria (0.0116 ±0.0077, 95%CI: -0.0034, 0.0266, p=0.1302). Metformin administration to older adults with glucose intolerance may be useful for frailty prevention and extension of healthspan.

